# Impact of Ultrasound–Assisted Method on Success Rate of Spinal Anesthesia Performed by Novice Trainees: A Retrospective Comparative Study

**DOI:** 10.3390/jpm13101515

**Published:** 2023-10-21

**Authors:** Antonio Coviello, Carmine Iacovazzo, Ilaria Piccione, Concetta Posillipo, Maria Silvia Barone, Marilena Ianniello, Andrea Uriel de Siena, Dario Cirillo, Maria Vargas

**Affiliations:** Department of Neurosciences, Reproductive and Odontostomatological Sciences, University of Naples “Federico II”, 80100 Naples, Italy; iacovazzo@tin.it (C.I.); ilariapiccione95@gmail.com (I.P.); concettaposillipo@libero.it (C.P.); baronemariasilvia@gmail.com (M.S.B.); marilena.ianniello@gmail.com (M.I.); andreauriel@outlook.it (A.U.d.S.); dariocirillo3@gmail.com (D.C.); vargas.maria82@gmail.com (M.V.)

**Keywords:** ultrasonography, spinal anesthesia, trainees, orthopedics, ultrasound-guided regional anesthesia

## Abstract

In current practice, single-shot spinal anesthesia has traditionally been performed using the conventional surface-anatomic-Landmark-Guided technique. This “blind” technique has significant critical issues such as a high risk of complications due to the numerous attempts at spinal needle placement and the negative impact on the learning curve of the trainees. Ultrasound-Assisted spinal anesthesia could reduce these critical issues and allow trainees to perform the procedure more easily and with fewer complications for the patient. We performed a before-and-after monocentric retrospective comparative study at the University of Naples “Federico II” (Naples, Italy). Inclusion criteria were as follows: patients aged 18 years or older; ASA physical status between I and IV; and elective orthopedic surgery under single-shot spinal anesthesia performed by supervised trainees between January 2022 and December 2022. In the selected cohort, 88 patients were included in group A (Landmark-Guided spinal anesthesia) and 91 in group B (Ultrasound-Assisted spinal anesthesia). The number of attempts by trainees (*p*-value < 0.005), procedure performing time (<0.001), and patient discomfort (<0.001) were significantly lower in group B than in group A. Ultrasound-Assisted single-shot spinal anesthesia performed by novice trainees reduces the number of attempts, complication rate, periprocedural pain, and patient discomfort.

## 1. Introduction

The ideal technique for spinal anesthesia is yet to be revealed and requires a successful dural puncture at the first attempt with the lowest possible discomfort for the patient. More punctures increase the risk of needle trauma, post-dural puncture headache, epidural hematoma, backache, paresthesia, and radicular post-procedural pain due to radicular puncture, and reduce the success rate of the procedure [1,2]. Spinal anesthesia has traditionally been performed using the well-known surface-Landmark-Guided (LG) technique. The intersection between the Tuffier’s line (the horizontal intercristal line) and the spinous process tips determines the needle insertion site. However, this ‘blind’ technique is often made more difficult to execute due to the following variables: anatomical variations, deformities, age-related degenerative changes, previous spinal surgery, and obesity; all of these variables may complicate the assessment of the correct spinal level for the puncture and increase the risk of multiple attempts for the same procedure [3,4,5,6]. Also, operators’ skills may significantly vary and affect the number of attempts and the success of the technique. Considering this, one of the factors associated with neuraxial damage or injury is the level of experience of the anesthesiologist [7]. To overcome these issues, the National Institute of Health and Clinical Excellence (NICE) in the United Kingdom has published guidelines suggesting that Ultrasound (US) could be used as a pre–procedural assessment tool for the anatomical variables and that it could be a guide during needle insertions [8]. The sonoanatomy of the column can be described in three approaches: sagittal, transverse, and diagonal [9]. In the sagittal view, the probe is located on the midline of the spinous process tips; the probe can then be tilted or laterally moved to obtain more scanning of the interlaminar level [9]. The transverse view can be used to assess the exact position of the interlaminar spaces; the operator can slide the probe caudally or cranially to determine the exact level of the dural location [9]. The diagonal view is used to perform real-time spinal anesthesia by visualizing the upper vertebral body, interlaminar space, and lamina of the lower vertebral body [9]. According to the above description, the US can be used to perform US-guided or US real-time spinal anesthesia. Specifically, US is increasingly used to guide spinal anesthesia and obtain detailed individual-specific anatomical information. The US helps to locate the midline, the intervertebral spaces, and their depth; these data allow the identification of the optimal needle insertion point, its appropriate orientation, and the location of the interlaminar anatomical window to permit the correct passage of the needle. The US-guided technique leads to a reduction in the number of puncture attempts and an increase in the success rate on the first attempt, even in patients with potentially difficult anatomy [10,11]. Many guidelines recommend using US guidance techniques to improve the efficacy, the safety, and the comfort of regional anesthesia procedures [12,13,14]. However, few data have been published to clarify the advantages and disadvantages of routine use of US for spinal anesthesia; indeed, it is still not clear which type of patient may benefit more from this technique [15,16]. In 2022, the University of Naples “Federico II” implemented such guidelines by introducing US in spinal anesthesia as the standard approach. To our knowledge, most of the studies dealt with teaching Ultrasound-Assisted (UA) spinal anesthesia, but none of them compared the use of US to improve trainees’ first-pass success rate with a “blind” technique in orthopedic patients using the ‘blind’ Landmark-Guided spinal anesthesia [17,18].

This study aims to fill these gaps in knowledge by investigating the impact on trainees’ learning curves of using the UA technique on orthopedic surgery patients. Specifically, the study aimed to compare the safety and efficacy of US-guided spinal anesthesia versus the LG technique performed by trainees. We hypothesized that US could help to reduce the number of skin punctures and the complication rate, increasing patient satisfaction and comfort.

## 2. Materials and Methods

Institutional ethical committee approval was not required since data were collected in daily clinical practice. All procedures were carried out in accordance with the 1964 Helsinki Declaration and its later amendments or comparable ethical standards. The Strengthening the Reporting of Observational Studies in Epidemiology (STROBE) statement was followed [19].

### 2.1. Study Design

This was a before-and-after monocentric retrospective comparative study performed at the Department of Surgical Sciences, Orthopedic Trauma, and Emergencies of the University of Naples “Federico II” (Naples, Italy) [20]. Data about patients who underwent spinal anesthesia at our institution between January 2022 and December 2022 were retrieved from the department archive then recorded on a pre-filled form and stored in a password-protected computerized database using Microsoft Office Excel.

The inclusion criteria were as follows: patients scheduled for elective orthopedic surgery of the lower limb with single-shot spinal anesthesia; aged 18 years or older; patients with a body mass index (BMI) of 18–40 kg/m^2^ based on the weight the day prior to surgery and the height measured at admission to the hospital; American Society of Anesthesiologists (ASA) physical status classification of I to IV; and anesthesia performed by a supervised novice trainee. “Novice trainees” are operators with less than one year of loco-regional anesthesia training and less than 20 spinal anesthesia procedures performed. The exclusion criteria were as follows: contraindications to subarachnoid anesthesia (i.e., infection at the puncture site, coagulopathy, allergy to the local anesthetic, or refusal of the procedure); patients on whom was performed combined spinal–epidural anesthesia or epidural anesthesia only; incomplete clinical data recorded; and pregnancy.

### 2.2. Anesthesiologic Management

All patients in the study received anesthetic management according to standard practices. In the operating room, peripheral venous access was in place (16–18 Gauge) and Pantoprazole 40 mg iv (intravenous), Ondansetron 8 mg iv, and antibiotic prophylaxis were administered (Cefazolin 1 or 2 g iv or—in the case of allergy—Clindamycin 600 mg iv) 30 min before skin incision. Perioperative management was performed according to institutional standards. Electrocardiogram (ECG), Continuous Non-Invasive Blood Pressure (NIBP), Pulse Oximetry (SpO2), and body temperature were monitored [21]. All patients were premedicated with 0.03 mg/kg iv Midazolam. All patients underwent spinal anesthesia using the LG (Group A) method (performed as standard procedure from January 2022 until May 2022 in the considered time period) or the UA (Group B) method (performed as standard procedure from June 2022 until December 2022 in the considered time period) in a sitting position. Strict asepsis was observed by the supervising anesthesiologist and the novice trainees, including hand hygiene, surgical mask, head scrub, sterile gloves, sterile gel, and sterile skin marker for group B.

In group A, spinal anesthesia was based on direct palpation of the surface anatomic landmarks (Tuffier’s line and the tips of spinous process) and the novice trainees used the midline approach. The procedure was performed as follows: firstly, the novice trainees palpated the iliac crests and the tips of the spinous processes; secondly, the Tuffier’s line was drawn between the iliac crests to find the body of L4 or the L4–L5 interspace; finally, the laminae were counted in the caudal-to-cephalic, or cephalad, direction until the desired level of spinal anesthesia was obtained; the novice trainees selected the interspinous space between L2 and L5 themselves.

In group B, before US imaging of the lumbar spine, the novice trainees selected the interspinous space between L2 and L5 by anatomic landmark palpation following the above-mentioned procedure. Then, the anesthesiologist, with notable experience (with at least five years of experience in ultrasound imaging of the spine, and with an average of more than 100 ultrasound imagings of the spine a year) performed ultrasound imaging of the lumbar spine with the 4C-RS convex probe (frequency, 2.0 to 5.5 MHz) of a portable ultrasound system (Sonosite HLF38 × 13–6 MHz, Fujifilm Sonosite Europe, Amsterdam, The Netherlands) and a regular-tip sterile skin marker. The probe was oriented longitudinally to obtain a parasagittal oblique view of the lumbosacral spine to count the interlaminar spaces upward from the sacrum to assess the desired level for the spinal anesthesia. Then, the probe was rotated by 90 degrees to obtain a transverse view of the lumbar spine; the skin was marked at the midpoints of the long and short borders of the probe. The intersection of these two marks and the probe angulation (left–right or cephalad) helped the novice trainees to perform the midline approach to spinal anesthesia. The ultrasound sterile gel was then wiped off to ensure that the needle-entry site was clean.

In both groups, anesthesia was administered using a 27 Gauge Whitacre spinal needle. After clear Cephalo-Spinal Fluid (CSF) was detected, Hyperbaric Bupivacaine 0.5% 10–12 mg, Sufentanil 5 μg, and Clonidine 30 μg as adjuvant, were injected.

The anthropometric parameters used to define difficulty in performing spinal anesthesia were as follows: high body mass index with challenging or impossible palpability of the spinous processes; visible deviations and the flexibility of the spine; previous spinal surgeries or multiple punctures for neuraxial procedures.

In both groups the novice trainees had three attempts after which the supervisor took over, given that more than three attempts increases the risk of spinal anesthesia failure.

### 2.3. Data Extraction

The following data were collected: demographic patient data (age, gender, and BMI), ASA, anatomical landmarks (palpability of the spinous processes and intercristal line), factors influencing column anatomy (scoliosis, previous spinal surgery, and BMI ≥ 30), and type of surgery and duration of the surgery procedure. Furthermore, we extracted data regarding the number of attempts (multiple redirections at one puncture level) performed by the novice trainees, number of procedures performed by the supervisor, the number of procedures performed by each novice trainee, interspace level of dural puncture, space identification time with LG or UA technique, and procedure performing time (from the insertion of the introducer needle to observing clear Cephalo-Spinal Fluid (CSF) in the spinal needle), total procedure time (space identification time, with LG or UA, plus procedure performing time), match between anatomical and echo-mediated landmarks, incidence of radicular pain, paresthesia, and blood aspirated by the spinal needle. Intraprocedural pain score was evaluated with a Numerical Rating Scale (NRS), whereby patients were asked to circle a number between 0 and 10. Zero usually represents “no pain at all” whereas the upper limit represents “the worst pain ever possible” [22]. Periprocedural discomfort (difficulty in maintaining position, pressure, or pushing, and paresthesia) was evaluated with a score from 0 to 4 (zero representing no discomfort, 1 mild discomfort, 2 moderate discomfort, 3 severe discomfort, and 4 extreme discomfort) [23].

### 2.4. Study Population

**Figure 1:**
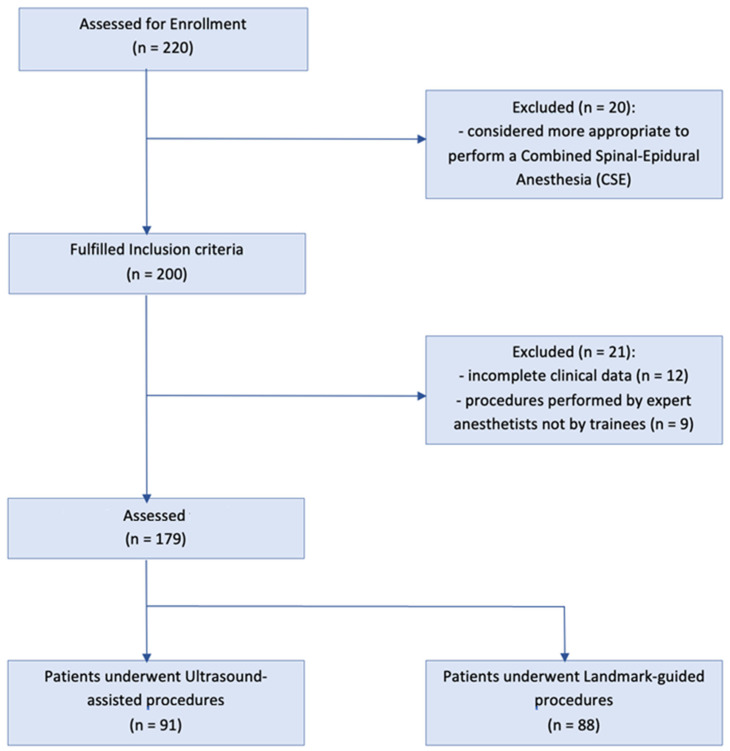
Study flowchart.

In the selected timeframe, 220 patients underwent elective orthopedic surgery with single-shot spinal anesthesia, performed by 20 novice trainees. After the application of exclusion criteria, 41 patients were excluded in the following way: 10 patients received a combined spinal–epidural or only epidural anesthesia; 9 patients were excluded because the anesthesiologist performed the spinal anesthesia at the first attempt; 10 patients were excluded because their procedures were performed by a trainee who did not meet the ‘novice trainee’ definition; and it was not possible to obtain the full clinical data of 12 patients). Finally, 179 patients were involved in the study and generated data for analysis (Figure 1). The patients were distributed as follows: 91 patients in group A, 88 patients in group B.

### 2.5. Statistical Analysis

Parametric data arere presented as mean and standard deviation, whereas non-parametric data are presented as data and interquartile range. Parametric data were analyzed using Student’s *t*-test and analysis of variance (ANOVA); non-parametric data were analyzed using the Mann–Whitney test and Friedman’s test. Categorical variables are presented as frequencies and a Pearson’s chi-square test was used to compare them.

After the first overall analysis, we conducted subgroup analyses using Student’s *t*-test for unpaired samples to correlate space identification time and procedure performing time to scoliosis.

All the tests were performed using the IBM SPSS software (version 28.0, IBM Corporation, New York, NY, USA).

We conducted a multivariate regression to evaluate the variables that influenced the procedure performing time using RStudio Team (version 2020, Integrated Development for R. RStudio, PBC, Boston, MA, USA). Statistical significance was set at *p*-value < 0.05.

## 3. Results

### 3.1. Patient Characteristics

In the selected cohort, 88 (49%) patients were included in group A and 91 (51%) patients in group B. No relevant difference in demographics, anatomical deformities of the spine or previous surgery, type of surgery, and duration of surgery between the two groups was observed (Table 1). Group A had more patients with no column abnormalities (36.4% vs. 17.6%, *p* = 0.005) but fewer obese individuals (37.5% vs. 61.5%, *p* = 0.005) and patients affected by scoliosis (66.6% vs. 82.4%, *p* = 0.005) than group B.

### 3.2. Intraand Periprocedural Details

The intra- and periprocedural details are reported in Table 2. The number of attempts needed by novice trainees to perform spinal anesthesia was significantly greater in group A than in group B (*p*-value 0.005). The differences in the number of attempts between the groups were as follows: 33.0% success at first attempt in group A against 57.1% in the group B (*p* = 0.001); 52.3% in group A against 33.0% in group B (*p* = 0.009); no significant difference in success at the first attempt. On the other hand, the senior operator had to take over 13 procedures in group A and 7 procedures in group B (*p* = 0.133). The periprocedural pain score was not significantly different between the two groups. Instead, patient discomfort during the procedure in group A was more significant than in group B (*p* < 0.001). The patients that felt ‘no discomfort’ or ‘mild discomfort’ were higher (*p* = 0.001) in group B (11% and 23.1%, respectively) in contrast with the patients of group A (0% and 9.1%, respectively); moreover, patients who felt ‘severe discomfort’ were more numerous (*p* = 0.002) in group A (28.4%) than in group B (9.9%).

### 3.3. Procedure Performing Time, Space Identification Time and Total Performing Time

**Figure 2:**
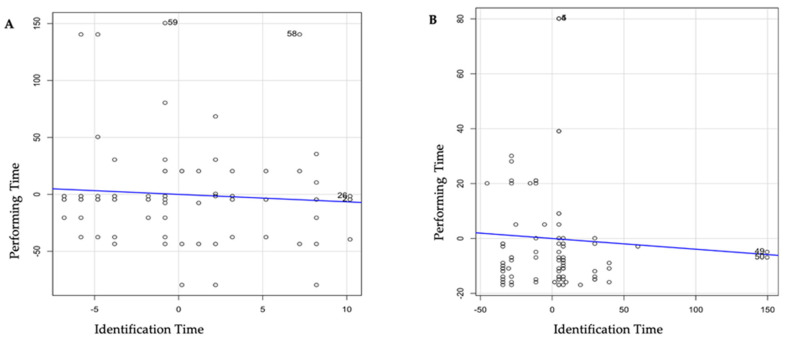
Linear Regression Model analyzing the relationship between procedure performing time and space identification time in the LG group (**A**) and the US group (**B**).

The time needed to perform spinal anesthesia (identifying landmarks and performing the procedure) was lower in the B group than in the A (99.5 ± 41.3 vs. 39.8 ± 22.3, *p* < 0.001) (Table 2), even considering the presence of factors influencing column anatomy such as scoliosis (Table 3). In group A, there was a difference between the patients affected by scoliosis and patients without scoliosis (48.5 ± 14.5 vs. 37.9 ± 23.2, *p* = 0.025) The incidence of radicular pain and paresthesia related to the spinal needle was 2/88 (2.27%) in group A versus 0/91 (0%) in group B. Figure 2 shows the linear regression between the performing time and identification time in groups A and B. In group B, higher identification time is related to a lower performing time, this correlation is not confirmed in group A. No difference was found in the total performing time between the evaluated groups.

### 3.4. Block Characteristics

The anatomical levels of spinal puncture reached by the anesthesiologic plane were similar in the two groups (Table 2). Table 2 shows the correspondence between anatomical and US-mediated landmarks in group B. The US-mediated landmarks corresponded to the anatomical procedure in 57 (62.6%) patients.

## 4. Discussion

This study aimed to validate the safety and efficacy of the educational possibilities of US-guided spinal anesthesia versus the LG technique, performed by novice trainees in patients with a difficult spine approach (such as patients with scoliosis, spinal deformities, and obesity), as often occurs in an orthopedic setting. So far, only a few studies have investigated different methods for teaching regional anesthesia to novice trainees, assessing the rate of success or comparing the discomfort, performing time, and complications between Ultrasound-Assisted spinal anesthesia and Landmark-Guided spinal anesthesia [24,25,26,27,28]. In this study, we analyzed the effects of diagnostic US imaging on success rates of spinal anesthesia and the risk of procedural complications. Our results showed that the US-assisted method performed by novice trainees in orthopedic surgery significantly reduced the number of attempts and passes of the needle with less periprocedural discomfort and complications than the traditional Landmark-Guided method. Additionally, the time needed to perform spinal anesthesia using the Landmark-Guided method was significantly higher than the time required using the UA method, probably due to the higher average number of attempts to perform the neuraxial procedure. The importance of this result is further confirmed by the fact that this analysis has been performed with patients having a very similar risk of procedure failure (scoliosis). Total procedure time was the same in both methods, suggesting that the use of ultrasound does not imply any significant delay in performing anesthesia in complicated patients.

Correct identification of interspinous space and the orientation of the immediately adjacent spinous process is essential for successful spinal anesthesia as it minimizes the number of attempts and the pain caused by multiple punctures, reducing the risk of spinal hematoma and post-dural puncture headache, and reducing the incidence of radicular pain, paresthesia, and patient discomfort [1,28,29]. Our results showed that the success rate of trainees in correctly identifying the subarachnoid space on their first attempt in orthopedic patients using the UA technique was 57.1%, unlike other studies that have shown a success rate of 88.4% [30]; the lower rate of h risk factors for anatomic distortion in the population of the other studies could explain the different result [30]. Furthermore, the mean number of attempts was significantly lower in the UA group than in the group using the conventional technique. The data given by the use of UA technique could explain the difference between the novice trainees in the two groups. Indeed, the UA technique gives the exact position of the interlaminar space and suggests the correct inclination of the needle to pass the yellow ligament; in this way, the novice trainee gains a lot of information that the LG technique can not reveal, and so can perform the procedure with more safety and efficacy. In contrast, Ansari et al. showed no significant difference in the total number of attempts; however, the characteristics of the cohorts included might explain this discrepancy since Ansari et al. selected only patients with easily palpable spines [31]. Our findings are similar to those of Perlas et al., who concluded that ultrasound is more accurate than palpation for the correct identification of lumbar interspaces and decreases the number of attempts required to perform the block [32]. It is important to underline that the learning of the trainee depends on multiple factors. According to other evidence, it is not only training that improves the learning curve of the trainee, but also the teaching skill and communication of the tutor and the motivation and knowledge of the trainee [32]. US provides a lot of anatomical information that could partially close the gap between the experience and knowledge of the anesthesiologist and the trainee.

Our results suggest that patients undergoing US-guided spinal anesthesia felt less discomfort compared to those undergoing the Landmark-Guided procedure. Other studies reported opposing results: if the comparison is between the LG and US-guided medial spinal procedures, there was no difference in the discomfort when the procedure is performed by an expert anesthesiologist; if the approach is paramedian, the group with US-guided procedure had lower levels of discomfort compared to the Landmark-Guided group [33,34]. This suggests that the discomfort of the patient could also be heavily influenced by the insertion point (median or paramedian) of the needle.

Multivariate linear regression analysis showed that the procedure performing time decreases in the US-guided group as the space identification time increases. Reducing procedural execution time decreases intraprocedural pain and periprocedural discomfort score, suggesting that more time for space identification with the Ultrasound-Assisted technique could ensure a more comfortable procedure for the patient. Interestingly, the analysis regarding the correlation between scoliosis/space identification time and scoliosis/procedure performing time showed that novice trainees took less time to perform the procedure using ultrasound, even in the presence of spinal abnormalities. This correlates with fewer attempts needed to perform dural puncture, reducing pain and discomfort for the patient. Space identification time is higher with the use of US, probably due to some technicism: the need to set the machine, the time to perform the ultrasonography, or the time to mark and trace the lines. Even if US is associated with higher identification time, the details obtained with this procedure increase the knowledge of the particular anatomy of the patients, helping to reduce the failure rate.

Our study found that the time taken to identify the interspinous space was lower in the LG method than in the UA method. This may be due to difficulty in identifying a satisfactory acoustic window during the use of ultrasound, especially in patients affected by scoliosis where the inclination and the rotation of the backbone could be difficult to evaluate. In spite of the identification time being longer in the UA group, the procedural time was significantly lower. Consequently, the total procedure time was the same between the two groups; this observation differs from that of Sahin et al., which showed that the total duration of the spinal procedure was shorter in the ultrasound group [35,36]. This could be explained by the correlation revealed by the multilinear regression: a higher identification time is related to a shorter performing time.

Finally, the decision to perform one method rather than another for the patients included in this cohort was completely down to the medical team.

This study is not free from limitations. Firstly, the study’s retrospective design reduces the generalizability of the results. The second limitation is the small number of patients included. Even if no power analysis was done at the beginning of the study, we believe that the lack of previous comparative analysis investigating the outcome of UA spinal anesthesia for teaching novice trainees increases the value of our results. Therefore, we advocate further prospective and comparative studies to confirm our findings and to clarify the real impact of US-guided spinal anesthesia.

Thirdly, there are no data on the posterior (ligamentum flavum, epidural space, and posterior dura) and anterior (anterior dura, posterior longitudinal ligament, and vertebral body) complexes, and the ultrasonographic estimation of depth to the subarachnoid space was not provided. This was arbitrarily hidden from trainees to let them develop greater technical skills in reaching the subarachnoid space but could affect the number of attempts of the trainees.

Fourthly, ultrasound imaging of the lumbar spine (in group B, in which the UA procedure was performed) was performed by an anesthesiologist with considerable experience. Since the assessment of the UA procedure was not among the aims of the study, this should not affect the reliability of our findings.

## 5. Conclusions

The Ultrasound-Assisted technique could represent a good and safe method to increase the success rate of single-shot spinal anesthesia in the elective orthopedic patient population, while allowing trainees to perform the procedure without complications due to the higher knowledge of the anatomy of the patient. It significantly reduces the number of attempts needed to perform spinal anesthesia, reducing patient intraprocedural pain and discomfort during the procedure. More studies are needed to clarify whether US spinal anesthesia has some advantages in terms of teaching and time and whether it could be a real alternative to performing spinal or other neuraxial procedures in elective surgical patients affected by illnesses that modify the anatomy of the spine.

## Figures and Tables

**Table 1:** SD (standard deviation), ASA (American Society of Anesthesiologists), BMI (body mass index), THR (total hip replacement arthroplasty), TKR (total knee replacement arthroplasty), rTKA (revision total knee arthroplasty), min (minutes). Data are expressed in mean ± standard deviation or number and percentage.

	Landmark-Guided (n = 88)	Ultrasound-Assisted (n = 91)	*p*-Value
Age (years)	82.5 ± 5.1	83 ± 5.8	0.562
Gender			0.237
Female	56 (63.9%)	50 (54.9%)	0.237
Male	32 (36.4%)	41 (45.1%)	0.237
BMI (kg/m^2^)	29.9 ± 4.9	31.1 ± 6.9	0.169
ASA physical status			0.383
1	0 (0%)	0 (0%)	1
2	42 (47.7%)	35 (38.5%)	0.211
3	44 (50.0%)	52 (57.1%)	0.338
4	2 (2.3%)	4 (4.4%)	0.430
Factors influencing column anatomy			0.005
None	32 (36.4%)	16 (17.6%)	0.005
Scoliosis	56 (63.6%)	75 (82.4%)	0.005
Previous spinal surgery	6 (6.8%)	11 (12.1%)	0.229
BMI ≥ 30	33 (37.5%)	56 (61.5%)	0.001
Type of surgery			0.686
THR	12 (13.6%)	11 (12.1%)	0.757
TKA	31 (35.2%)	26 (28.6%)	0.339
rTKA	36 (40.9%)	41 (45.1%)	0.575
Ankle or foot surgery	9 (10.2%)	13 (14.3%)	0.408
Duration of surgery (min)	87.3 ± 19.4	88.5 ± 20.6	0.702

**Table 2:** SD (standard deviation), NRS (Numerical Rating Scale), s (seconds), * not valuable. Data are expressed in mean ± standard deviation or number and percentage.

	Landmark-Guided (n = 88)	Ultrasound-Assisted (n = 91)	*p*-Value
Attempts performed by novice trainees			0.005
1	29 (33.0%)	52 (57.1%)	0.001
2	46 (52.3%)	30 (33.0%)	0.009
3	13 (14.8%)	9 (9.9%)	0.320
Procedures performed by tutor	13 (14.8%)	7 (7.7%)	0.133
Procedure performed by each novice trainee	4.7 ± 1.2	4.3 ± 1.1	0.066
Interspace level of dural puncture			0.138
L2–L3	73 (83.0%)	68 (74.7%)	0.178
L3–L4	10 (11.4%)	20 (22.0%)	0.057
L4–L5	5 (5.7%)	3 (3.3%)	0.440
L5–S1	0 (0.0%)	0 (0.0%)	*
Space identification time (s)	29.7 ± 5.1	90.3 ± 31.2	<0.001
Procedure performing time (s)	99.5 ± 41.3	39.8 ± 22.3	<0.001
Total procedure time (s)	123.4 ± 31.9	124.5 ± 31.9	0.818
Match between anatomical and echo-mediated landmarks			*
Match			
No match		57 (62.6%)	
Complications		34 (37.4%)	
Incidence of radicular pain	1 (1.1%)	0 (0.0%)	0.308
Paresthesia by the spinal needle	2 (2.3%)	0 (0.0%)	0.148
Blood tap by the spinal needle	0 (0.0%)	0 (0.0%)	*
Periprocedural pain score (NRS)	3.8 ± 1.6	3.7 ± 1.7	0.608
Periprocedural discomfort score			<0.001
0 (no discomfort)	0 (0.0%)	10 (11.0%)	0.001
1 (no mild discomfort)	8 (9.1%)	21 (23.1%)	0.011
2 (moderate discomfort)	39 (44.3%)	42 (46.2%)	0.805
3 (severe discomfort)	25 (28.4%)	9 (9.9%)	0.002
4 (extreme discomfort)	16 (18.2%)	9 (9.9%)	0.110

**Table 3:** Sub-group analysis—Unpaired samples t-test between scoliosis and space identification time and between scoliosis and procedure performing time in the Landmark-Guided group (a) and in the Ultrasound-Assisted group (b). SD (standard deviation), s (seconds).

**(a) Landmark-Guided** **(n = 88)**		
	**Mean ± SD**	***p*-Value**
Space identification time (s)		0.913
without scoliosis	29.6 ± 4.9	
with scoliosis	29.3 ± 5.3	
Procedure performing time (s)		0.538
without scoliosis	103.5 ± 50.1	
with scoliosis	97.3 ± 35.6	
**(b) Ultrasound-Assisted ** **(n = 91)**		
	**Mean ± SD**	***p*-Value**
		0.067
Space identification time (s)	82.1 ± 14.9	
without scoliosis	92.1 ± 33.5	
with scoliosis		
Procedure performing time (s)		0.025
without scoliosis	48.5 ± 14.5	
with scoliosis	37.9 ± 23.2	

## Data Availability

Data is contained within the article.
